# Supplementary material for: Phosphorylation of muramyl peptides by NAGK is required for NOD2 activation
Source: Nature. 2022 Aug 24;609(7927):590–6. doi: 10.1038/s41586-022-05125-x (PMC9477735; doi:10.1038/s41586-022-05125-x)
Supplement: Supplementary file 1 — This file contains Supplementary Methods and Supplementary Figs. 1-2 [file 41586_2022_5125_MOESM1_ESM.pdf]

nature portfolio

https://doi.org/10.1038/s41586-022-05125-x

Supplementary information

Phosphorylation of muramyl peptides by NAGK is required for NOD2 activation

In the format provided by the authors and unedited

Supplementary Methods. Synthesis and analytic details for MurNAc-Ala-D-isoGln-Lys-Fluorescein (MTP-Fluorescein) and lac-Ala-D-isoGln-Lys-Fluorescein (lac-TP-Fluorescein)

Mass spectrometry

Mass spectrometry analysis was done on a Q Exactive Plus mass spectrometer (Thermo Scientific, Bremen, Germany) using a Triversa Nanomate (Advion, Ithaca, NY) as ion source. All measurements were performed in negative-ion mode using a spray voltage of -1.1 kV. Samples were dissolved in a water/propan-2-ol/trimethylamine/acetic acid mixture (50:50:0.06:0.02, v/v/v/v) in a final concentration of 0.10 mg ml^{-1}. All mass spectra were charge deconvoluted and given mass values refer to the monoisotopic mass of the neutral molecules, if not indicated otherwise. Deconvoluted spectra were computed using Xtract module of Xcalibur 3.1. Software (Thermo, Bremen, Germany).

NMR spectroscopy

NMR spectroscopic measurements were performed at 300 K on a Bruker Avance^{III} 700 MHz spectrometer equipped with an inverse 5 mm quadruple-resonance Z-grad cryoprobe (spectrometer frequencies: 700.43 MHz for ^{1}H, 176.12 MHz for ^{13}C). Acetone was used as an external standard for calibration of ^{1}H (δ_H = 2.225 ppm) and ^{13}C (δ_C = 30.89 ppm) NMR spectra. All data were acquired and processed by using Bruker TOPSPIN V 3.1 or higher (Bruker BioSpin Corporation, Billerica, MA, USA). The parameter sets used were adapted starting from respective Bruker standard parameter sets, which are all included in this software. Prior to analysis the compounds were repeatedly exchanged in deuterated water (D_2O; 99.98%, Deutero GmbH, Kastellaun, Germany) with intermediate lyophilisation. ^{1}H NMR assignments were confirmed by 2D ^{1}H,^{1}H-COSY and -TOCSY experiments, ^{13}C NMR assignments were indicated by 2D ^{1}H,^{13}C-HSQC, based on the ^{1}H NMR assignments.

Special chemicals and compounds

MurNAc-Ala-D-isoGln-Lys (M-Tri Lys, Catalog Code: #tlrl-mtl) was purchased from Invivogen, NHS Fluorescein from Thermo Scientific (Catalog Code: 46409). Methanol (SupraSolv®) for HPLC and N,N-dimethylformamide (DMF; Uvasol®) were obtained from Merck, acetonitrile (Rotisolv®) for HPLC from Roth, trifluoroacetic acid (TFA; Uvasol®) from Supelco.

HPLC system and method

For HPLC purifications the following system has been used: *company*: Gilson; *pump*: model -305/ -306; *interface*: 811D; *detection*: UV/VIS-151; *degasser*: phenomenex model DG-4400; *fraction collector*: FC203B; *software*: Unipoint 3.30. As a preparative column the Macherey-Nagel NUCLEODUR C_{18} Pyramid 5 µm, 21 x 250 mm was used, as a pre-column the Zorbax ODS 5 µm, 10

x 4.0 mm column was used. The gradient was run as follows: H_2O (0.05% TFA)/acetonitrile (0.05% TFA) 99:1 v/v from 0 to 10 min, then H_2O (0.05% TFA) with an acetonitrile (0.05% TFA) gradient (1-50%) until 120 min, then H_2O (0.05% TFA)/acetonitrile (0.05% TFA) 50:50 v/v until 142 min, the column was re-equilibrated during 13 min to H_2O (0.05% TFA)/acetonitrile (0.05% TFA) 99:1 v/v and held there for additional 10 min, flow 2.5 ml min^{-1}, UV detection at 206 nm.

Synthesis of MurNAc-Ala-D-isoGln-Lys-Fluorescein (MTP-Fluorescein)

MurNAc-Ala-D-isoGln-Lys (M-Tri Lys) was dissolved in 80 mM K_2HPO_4/KH_2PO_4-buffer (pH 8.0) (appr. 1.3 µmol ml^{-1}). A 10 mg ml^{-1} solution of NHS Fluorescein in the same buffer but containing 5% DMF was freshly prepared and a portion containing two equivalents NHS Fluorescein compared to M-Tri Lys was added. The resulting reaction mixture was stirred for 1 h at 37 °C. After cooling to RT, this solution was directly applied to a self-packed RP18-column (15 x 40 mm; Waters preparative C_{18} 125Å 55-105 µm), which was washed with 10 ml double distilled water/methanol (1:1 v/v) and afterwards equilibrated with 10 ml double distilled water prior usage. As eluents were used: 1) 50 ml double distilled water, 2) 50 ml double distilled water/methanol (1:1 v/v), and 3) 25 ml methanol. The target compound eluted in the water/methanol (1:1 v/v)-fraction, which was concentrated under reduced pressure and afterwards purified by preparative HPLC (Fig. S1). For injection, the material was dissolved in 2 ml double distilled water.

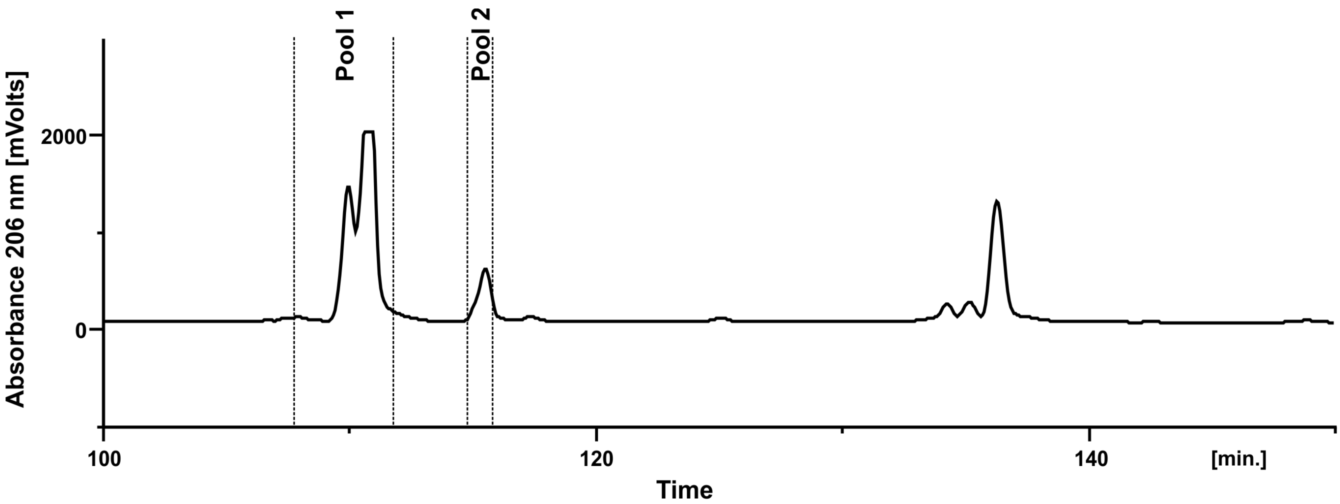

Figure S1. Representative chromatogram of the reversed-phase HPLC purification (section shown: 100 to 150 min), pooled fractions are indicated.

Indicated pools were collected and freeze-dried in a Scanvac CoolSafe 110-4 Pro (Scanlaf). Resulting residues were dissolved in acetonitrile/water (1:1 v/v), freeze-dried a second time and afterwards weighted. Structural analysis by NMR and MS was performed as described above, for analytical data see below. Pool 1 contained the target compound MurNAc-Ala-D-isoGln-Lys-Fluorescein (MTP-

Fluorescein), pool 2 contained the reaction side product lactoyl-Ala-D-isoGln-Lys-Fluorescein (lac-TP-Fluorescein), which lacks the GlcNAc moiety of the MurNAc.

A reaction of 530 µg (0.854 µmol) M-Tri Lys in 645 µl 80 mM K_2HPO_4/KH_2PO_4-buffer (pH 8.0) with 809 µg (1.71 µmol) NHS Fluorescein in 80.9 µl 80 mM K_2HPO_4/KH_2PO_4-buffer (pH 8.0)/DMF (19:1, v/v) yielded 580 µg (0.592 µmol, 69%) MTP-Fluorescein and 200 µg (0.258 µmol, 30%) lac-TP-Fluorescein.

In a second synthesis, a reaction of 560 µg (0.902 µmol) M-Tri Lys in 681 µl 80 mM K_2HPO_4/KH_2PO_4-buffer (pH 8.0) with 854 µg (1.81 µmol) NHS Fluorescein in 85.4 µl 80 mM K_2HPO_4/KH_2PO_4-buffer (pH 8.0)/DMF (19:1, v/v) yielded 420 µg (0.429 µmol, 48%) MTP-Fluorescein and 90 µg (0.116 µmol, 13%) lac-TP-Fluorescein.

Analytical data for MTP-Fluorescein

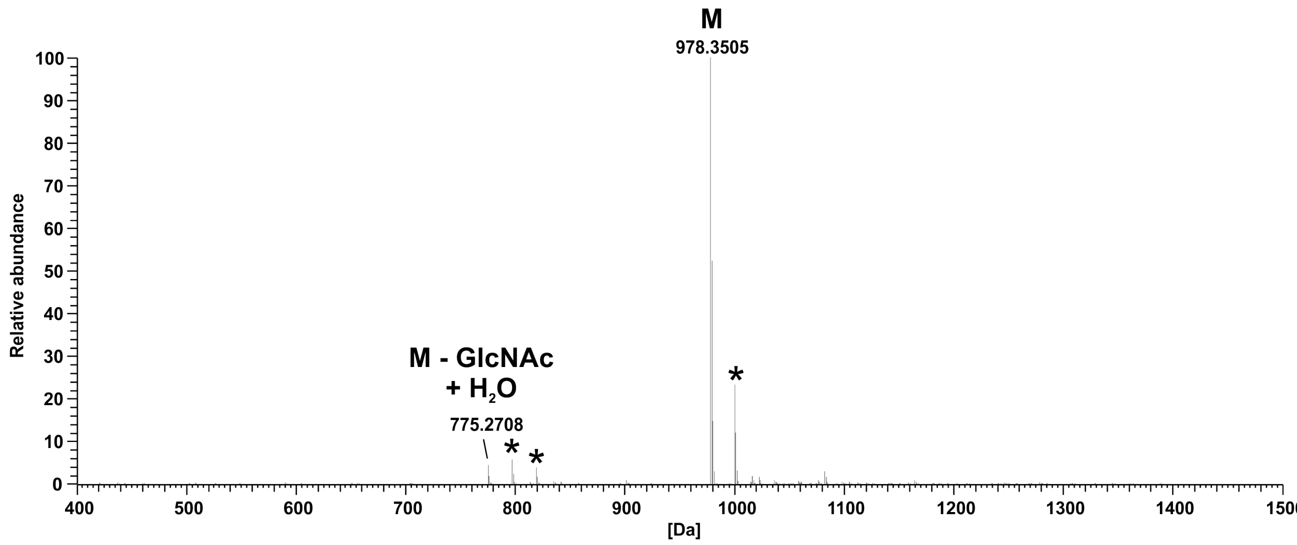

Figure S2. Charge-deconvoluted spectrum of a representative MS analysis of MTP-Fluorescein (pool 1 of Fig. S1); calculated exact mass: 978.3495 Da, observed mass: 978.3505 Da. Signals labelled with * represent sodium adducts (Δm = +21.98 Da).

^{1}H NMR (700.43 MHz, D_2O): δ = 8.18 (s, 2 x 1H, aryl-H), 7.96 (d, J = 8.0 Hz, 2 x 1H, aryl-H), 7.47 (d, J = 8.0 Hz, 2x 1H, aryl-H), 7.18 (d, J = 9.0 Hz, 2 x 2H, aryl-H), 6.67-6.63 (m, 4 x 2H aryl-H), 5.12 (d, J = 3.3 Hz, 1H, H-1α_{MurNAc}), 4.66 (d, J = 8.5 Hz, 1H, H-1β_{MurNAc}), 4.30-4.24 (m, 2 x 1H, H-2α_{iGln}, H-2β_{iGln}), 4.28-4.23 (m, 2 x 1H, CHα_{Ala}, CHβ_{Ala}), 4.30-4.25 (m, 1H, C*H*$CH_3\alpha_{MurNAc}$), 4.21-4.17 (m, 2 x 1H, H-2α_{Lys}, H-2β_{Lys}), 4.18-4.14 (m, 1H, C*H*$CH_3\beta_{MurNAc}$), 3.92 (dd, J = 10.5, 3.3 Hz, 1H, H-2α_{MurNAc}), 3.91-3.87 (m, 1H, H-6aβ_{MurNAc}), 3.87-3.83 (m, 1H, H-5α_{MurNAc}), 3.84-3.80 (m, 1H, H-6aα_{MurNAc}), 3.80-3.76 (m, 1H, H-6bα_{MurNAc}), 3.78-3.74 (m, 1H, H-2β_{MurNAc}), 3.76-3.72 (m, 1H,

H-6bβ_{MurNAc}), 3.71-3.66 (m, 1H, H-3α_{MurNAc}), 3.57-3.52 (m, 1H, H-4α_{MurNAc}), 3.54-3.49 (m, 1H, H-4β_{MurNAc}), 3.49-3.45 (m, 1H, H-3β_{MurNAc}), 3.47-3.43 (m, 1H, H-5β_{MurNAc}), 3.49-3.45 (m, 2 x 2H, H-6α_{Lys}, H-6β_{Lys}), 2.42-2.36 (m, 2 x 2H, H-4$\alpha_{i\text{Gln}}$, H-4$\beta_{i\text{Gln}}$), 2.18-2.12 (m, 2 x 1H, H-3a$\alpha_{i\text{Glu}}$, H-3a$\beta_{i\text{Glu}}$), 1.94 (s, 3H, $\text{COCH}_{3\text{MurNAc}}$), 1.93 (s, 3H, $\text{COCH}_{3\text{MurNAc}}$), 2.01-1.97 (m, 2 x 1H, H-3b$\alpha_{i\text{Glu}}$, H-3b$\beta_{i\text{Glu}}$), 1.90-1.85 (m, 2 x 1H, H-3aα_{Lys}, H-3aβ_{Lys}), 1.76-1.71 (m, 2 x 1H, H-3bα_{Lys}, H-3bβ_{Lys}), 1.71-1.66 (m, 2 x 2H, H-5α_{Lys}, H-5β_{Lys}), 1.50-1.44 (m, 2 x 2H, H-4α_{Lys}, H-4β_{Lys}), 1.43-1.36 (m, 2 x 3H, $\text{CH}_3\alpha_{\text{Ala}}$, $\text{CH}_3\beta_{\text{Ala}}$), 1.34-1.32 (m, 3H, $\text{CH}C\!H_3\alpha_{\text{MurNAc}}$), 1.32-1.30 (m, 3H, $\text{CH}C\!H_3\beta_{\text{MurNAc}}$) ppm.

^{13}C NMR (176.12 MHz, D_2O): δ = 131.9 (aryl-C), 131.1 (aryl-C), 128.4 (aryl-C), 127.4 (aryl-C), 123.5 (aryl-C), 104.0 (aryl-C), 95.5 (C-1β_{MurNAc}), 91.5 (C-1α_{MurNAc}), 83.0 (C-3β_{MurNAc}), 80.0 (C-3α_{MurNAc}), 78.5 ($C\text{HCH}_3\beta_{\text{MurNAc}}$), 78.2 ($C\text{HCH}_3\alpha_{\text{MurNAc}}$), 76.3 (C-5$\beta_{\text{MurNAc}}$), 72.1 (C-5$\alpha_{\text{MurNAc}}$), 69.7 (C-4$\alpha_{\text{MurNAc}}$), 69.4 (C-4$\beta_{\text{MurNAc}}$), 61.3 (C-6$\beta_{\text{MurNAc}}$), 61.1 (C-6$\alpha_{\text{MurNAc}}$), 56.6 (C-2$\beta_{\text{MurNAc}}$), 54.2 (C-2$\alpha_{\text{MurNAc}}$), 55.8 (C-2$\alpha_{\text{Lys}}$, C-2$\beta_{\text{Lys}}$), 53.6 (C-2$\alpha_{i\text{Gln}}$, C-2$\beta_{i\text{Gln}}$), 50.3 (CH$\alpha_{\text{Ala}}$, CH$\beta_{\text{Ala}}$), 40.5 (C-6$\alpha_{\text{Lys}}$, C-6$\beta_{\text{Lys}}$), 32.5 (C-4$\alpha_{i\text{Gln}}$, C-4$\beta_{i\text{Gln}}$), 31.9 (C-3$\alpha_{\text{Lys}}$, C-3$\beta_{\text{Lys}}$), 28.7 (C-5$\alpha_{\text{Lys}}$, C-5$\beta_{\text{Lys}}$, 27.6 (C-3$\alpha_{i\text{Gln}}$, C-3$\beta_{i\text{Gln}}$), 23.5 (C-4$\alpha_{\text{Lys}}$, C-4$\beta_{\text{Lys}}$), 22.6 ($\text{CO}C\text{H}_3\alpha_{\text{MurNAc}}$, $\text{CO}C\text{H}_3\beta_{\text{MurNAc}}$), 19.2 ($\text{CH}C\text{H}_3\alpha_{\text{MurNAc}}$, $\text{CH}C\text{H}_3\beta_{\text{MurNAc}}$), 17.2 ($\text{CH}_3\alpha_{\text{Ala}}$, $\text{CH}_3\beta_{\text{Ala}}$) ppm. (Chemical shifts for quaternary carbons are not stated since detection or unequivocal assignment was not possible due to limited sample amount)

Mass spectrometry data for lac-TP-Fluorescein

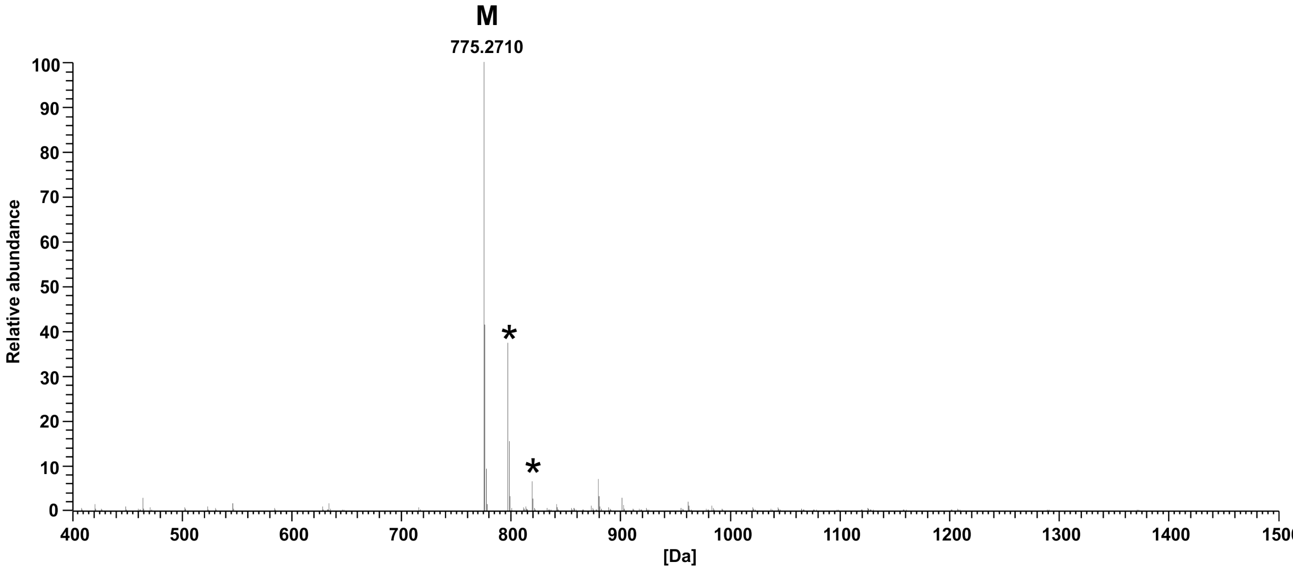

Figure S3. Charge-deconvoluted spectrum of a representative MS analysis of lac-TP-Fluorescein (pool 2 of Fig. S1); calculated exact mass: 775.2701 Da, observed mass: 775.2710 Da. Signals labelled with * represent sodium adducts (Δm = +21.98 Da).

Fig. 2a

membrane a

IB:NAGK

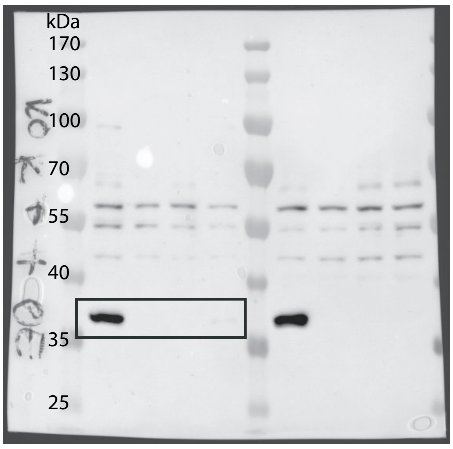

membrane a

IB: β-actin

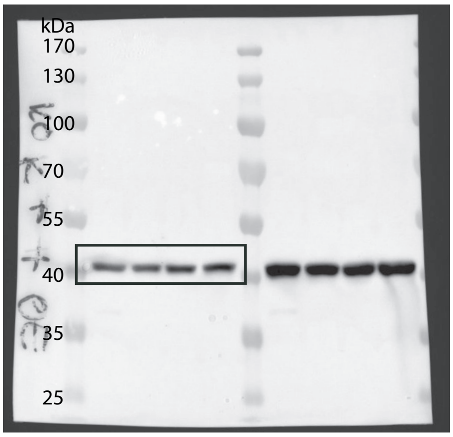

Fig. 2c

membrane b

IB:NAGK

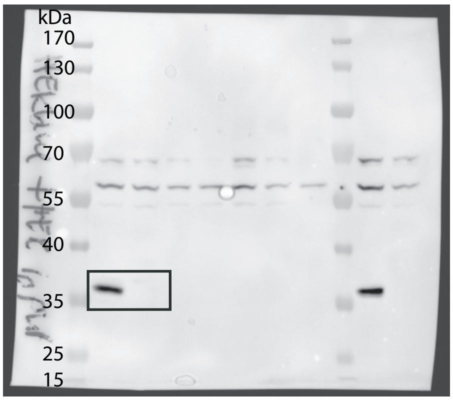

membrane b

IB: β-actin

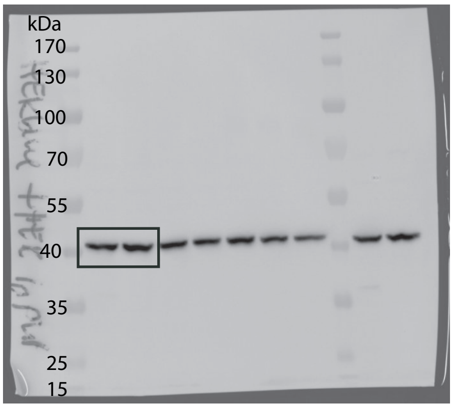

Fig. 2i

membrane b (same image as above)

IB:NAGK

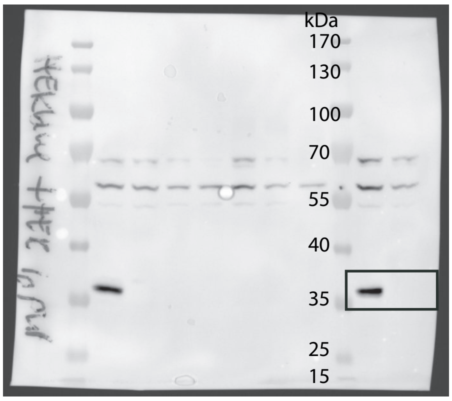

membrane b (same image as above)

IB: β-actin

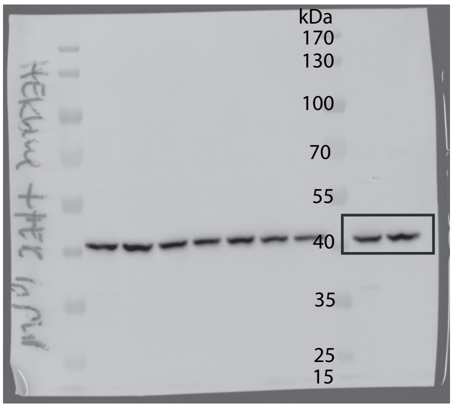

Supplementary Figure 1. Uncropped blot source data

Uncropped immunoblot images are shown, while the corresponding figures are indicated for each set of blots. The lines indicate the cropping margins. In order to display the size marker, a merged image of the image derived from the chemiluminescent signal and the image derived from the white-light epi-illumination signal is depicted. Some membranes were re-probed with another antibody. To illustrate what membranes were re-probed, each membrane is labelled above.

Fig. 2f

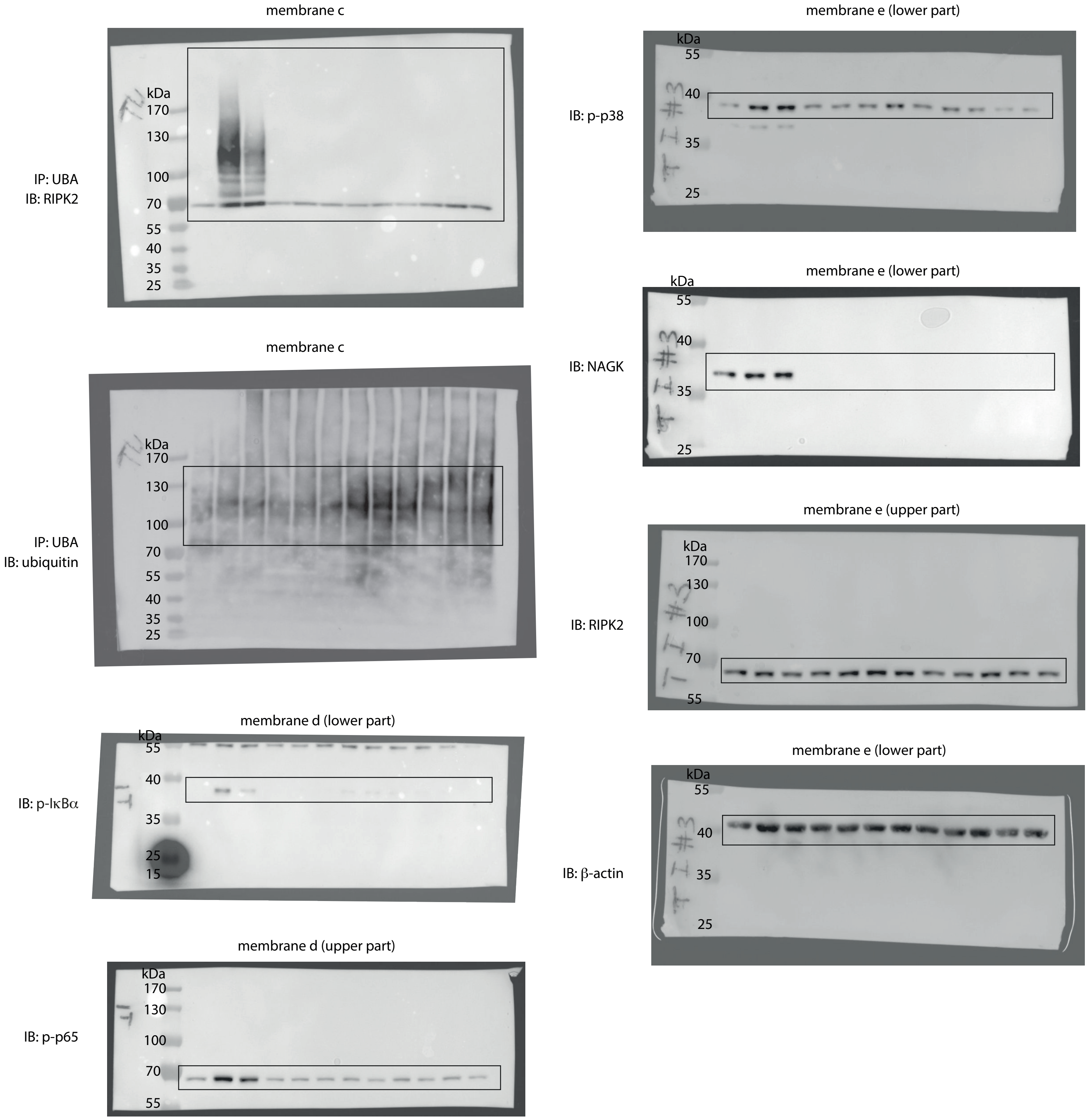
membrane c
IP: UBA
IB: RIPK2
kDa
170
130
100
70
55
40
35
25
membrane c
IP: UBA
IB: ubiquitin
kDa
170
130
100
70
55
40
35
25
membrane d (lower part)
IB: p-IκBα
kDa
55
40
35
25
15
membrane d (upper part)
IB: p-p65
kDa
170
130
100
70
55
membrane e (lower part)
IB: p-p38
kDa
55
40
35
25
membrane e (lower part)
IB: NAGK
kDa
55
40
35
25
membrane e (upper part)
IB: RIPK2
kDa
170
130
100
70
55
membrane e (lower part)
IB: β-actin
kDa
55
40
35
25

Fig. 4c

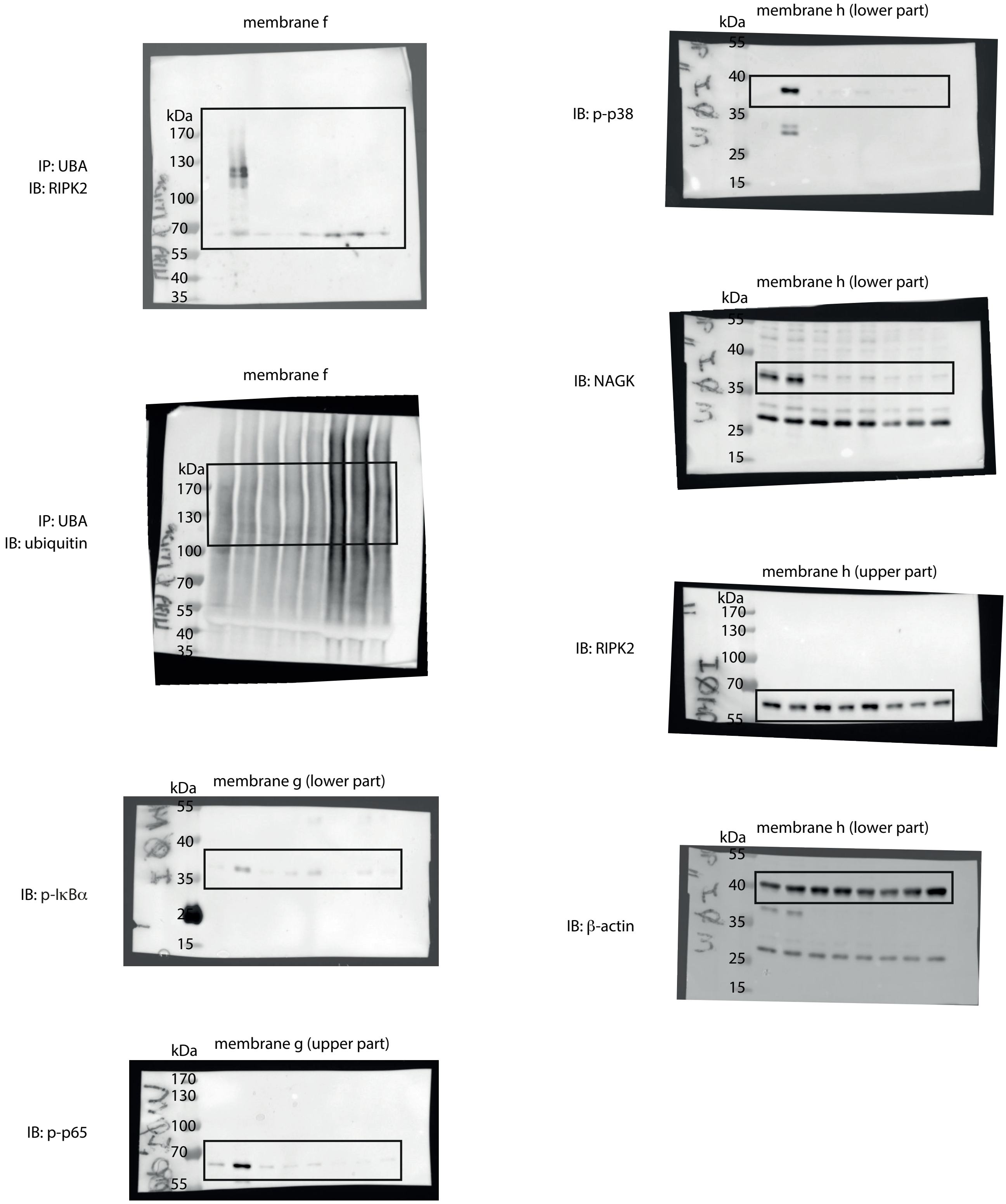

membrane f
IP: UBA
IB: RIPK2
kDa
170
130
100
70
55
40
35
membrane f
IP: UBA
IB: ubiquitin
kDa
170
130
100
70
55
40
35
membrane g (lower part)
IB: p-IκBα
kDa
55
40
35
25
15
membrane g (upper part)
IB: p-p65
kDa
170
130
100
70
55
membrane h (lower part)
IB: p-p38
kDa
55
40
35
25
15
membrane h (lower part)
IB: NAGK
kDa
55
40
35
25
15
membrane h (upper part)
IB: RIPK2
kDa
170
130
100
70
55
membrane h (lower part)
IB: β-actin
kDa
55
40
35
25
15

Extended Data Fig. 5c

membrane a

IB: NAGK

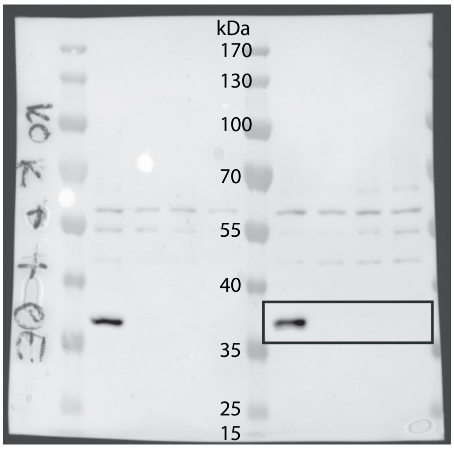

membrane a

IB: β-actin

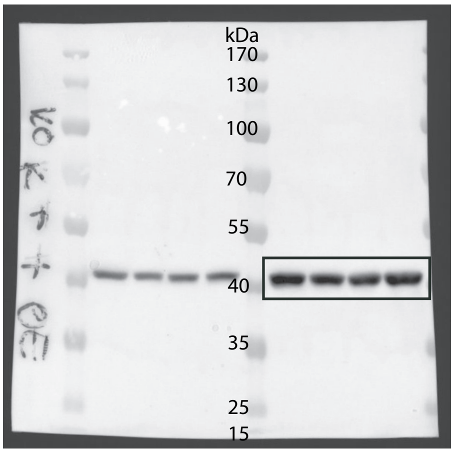

Extended Data Fig. 5e (first part)

membrane i

IP: UBA
IB: RIPK2

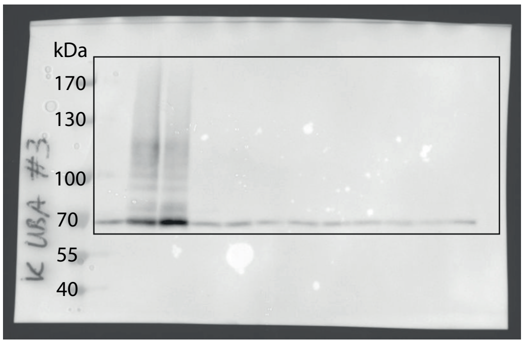

membrane i

IP: UBA
IB: ubiquitin

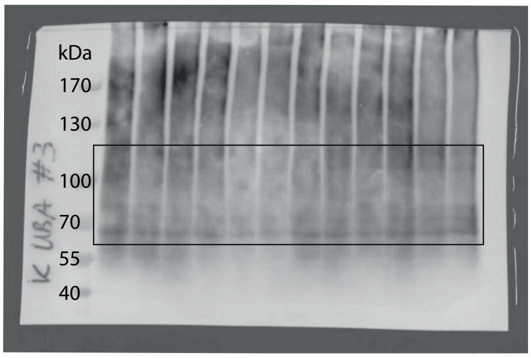

Extended Data Fig. 5e (second part)

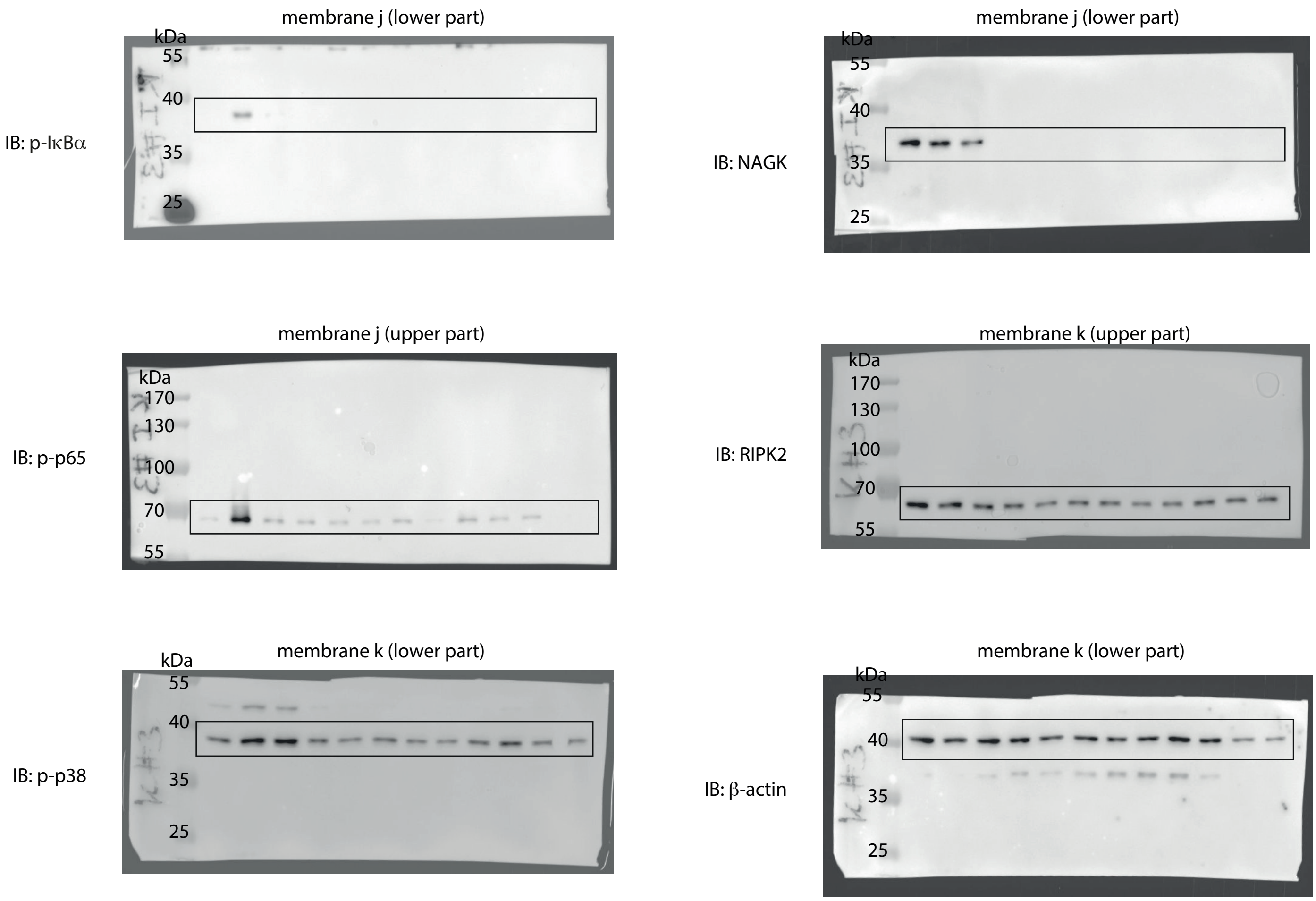

Extended Data Fig. 7a

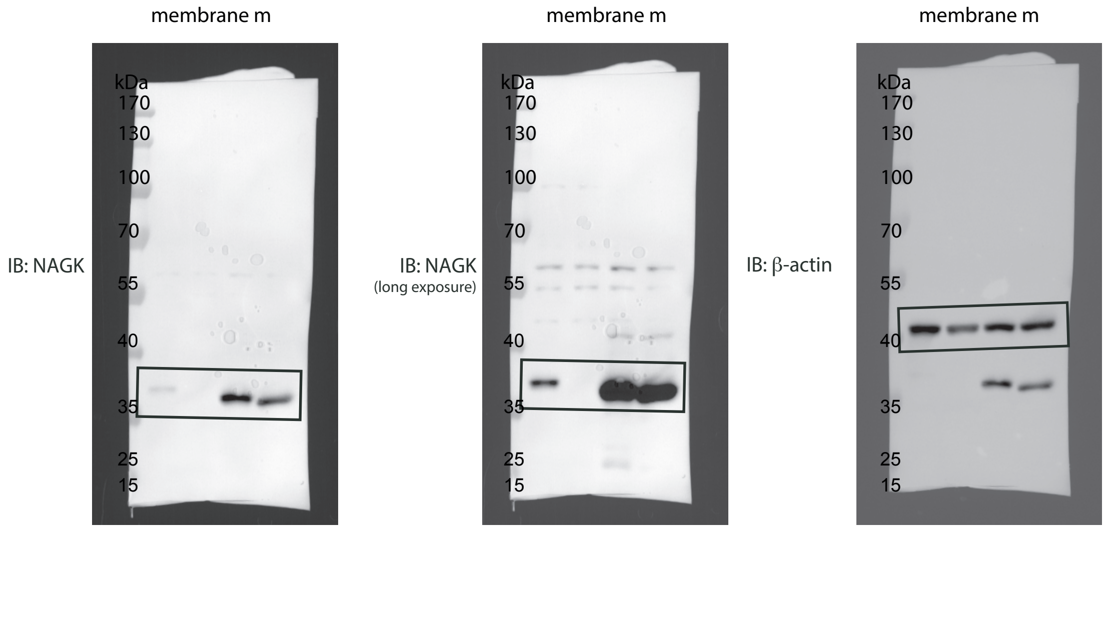

a

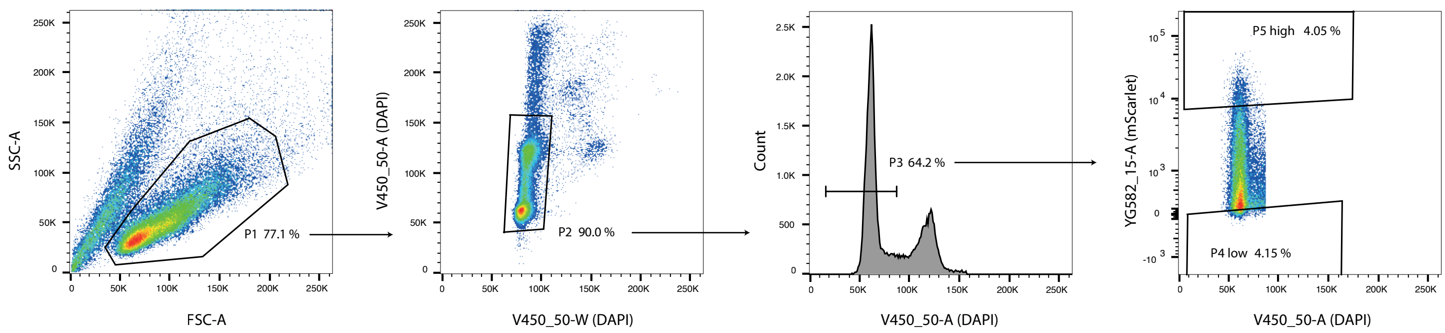

b

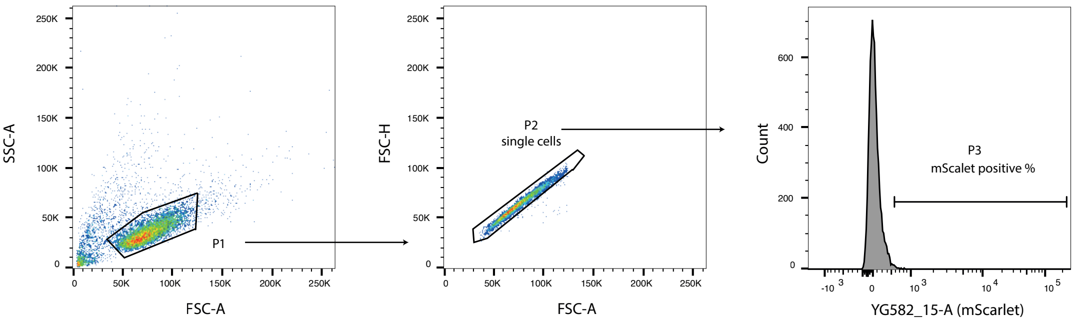

Supplementary Figure 2. Flow cytometry gating strategy

a, The flow cytometry gating strategy for the KBM-7 haploid genetic screen for regulators of NOD2 is depicted. **b**, The flow cytometry gating strategy to identify mScarlet-positive cells for the KBM-7 stimulation assays is depicted.
