# Supplementary material for: Phosphorylation of muramyl peptides by NAGK is required for NOD2 activation
Source: Nature. 2022 Aug 24;609(7927):590–6. doi: 10.1038/s41586-022-05125-x (PMC9477735; doi:10.1038/s41586-022-05125-x)
Supplement: Supplementary file 2 — Reporting Summary [file 41586_2022_5125_MOESM2_ESM.pdf]

nature portfolio

Corresponding author(s): Veit Hornung

Last updated by author(s): Jun 25, 2022

Reporting Summary

Nature Portfolio wishes to improve the reproducibility of the work that we publish. This form provides structure for consistency and transparency in reporting. For further information on Nature Portfolio policies, see our Editorial Policies and the Editorial Policy Checklist.

Statistics

For all statistical analyses, confirm that the following items are present in the figure legend, table legend, main text, or Methods section.

n/a	Confirmed	
☐	☒	The exact sample size (n) for each experimental group/condition, given as a discrete number and unit of measurement
☐	☒	A statement on whether measurements were taken from distinct samples or whether the same sample was measured repeatedly
☐	☒	The statistical test(s) used AND whether they are one- or two-sided<br>*Only common tests should be described solely by name; describe more complex techniques in the Methods section.*
☒	☐	A description of all covariates tested
☐	☒	A description of any assumptions or corrections, such as tests of normality and adjustment for multiple comparisons
☐	☒	A full description of the statistical parameters including central tendency (e.g. means) or other basic estimates (e.g. regression coefficient) AND variation (e.g. standard deviation) or associated estimates of uncertainty (e.g. confidence intervals)
☐	☒	For null hypothesis testing, the test statistic (e.g. F, t, r) with confidence intervals, effect sizes, degrees of freedom and P value noted<br>*Give P values as exact values whenever suitable.*
☒	☐	For Bayesian analysis, information on the choice of priors and Markov chain Monte Carlo settings
☒	☐	For hierarchical and complex designs, identification of the appropriate level for tests and full reporting of outcomes
☐	☒	Estimates of effect sizes (e.g. Cohen's d, Pearson's r), indicating how they were calculated

Our web collection on statistics for biologists contains articles on many of the points above.

Software and code

Policy information about availability of computer code

Data collection

For data collection the following software was used: BD FACSDiva 8.0.1 and 8.0.2.

Data analysis

All statistical analysis were performed using GraphPad Prism 9.

Flow cytometry data were analysed using FlowJo 10.7.

For correlation analysis, analysis and plotting were performed in R (version 4.1.0.). Pearson correlation matrix was calculated using base R function cor(). Genes were ordered by their correlation coefficient to NAGK, and the cut-off was made at r ≥ 0.7 (R code for analysis and plotting is available upon request).

Gene ontology enrichment analysis was done using PANTHER GO-slim.

Mass spec raw files were processed by the Spectronaut software version 14 using the directDIA option.

For manuscripts utilizing custom algorithms or software that are central to the research but not yet described in published literature, software must be made available to editors and reviewers. We strongly encourage code deposition in a community repository (e.g. GitHub). See the Nature Portfolio guidelines for submitting code & software for further information.

Data

Policy information about availability of data

All manuscripts must include a data availability statement. This statement should provide the following information, where applicable:

- Accession codes, unique identifiers, or web links for publicly available datasets
- A description of any restrictions on data availability
- For clinical datasets or third party data, please ensure that the statement adheres to our policy

All data including Source Data for Figs. 1–4 and Extended Data Fig. 3-7 and 9 and 10 are provided with the paper and its Supplementary Information files.

The MS-based proteomics data have been deposited to the ProteomeXchange Consortium via the PRIDE partner repository and are available via the identifier PXD022384. https://www.ebi.ac.uk/pride/archive/projects/PXD022384

Deep-sequencing raw data (genome-wide genetic screen) have been deposited in the NCBI Sequence Read Archive under accession number PRJNA841795. https://www.ncbi.nlm.nih.gov/bioproject/PRJNA841795
The corresponding processed data are provided in Supplementary Table 1.

Raw LC-MS data have been submitted to MassIVE and can be accessed with ID MSV000088170. http://dx.doi.org/10.25345/C58861

R code for analysis and plotting is available at Github: https://github.com/Pestudkaru/Corr_analysis.

Materials and reagents are available from the corresponding author upon request.

Field-specific reporting

Please select the one below that is the best fit for your research. If you are not sure, read the appropriate sections before making your selection.

☒ Life sciences ☐ Behavioural & social sciences ☐ Ecological, evolutionary & environmental sciences

For a reference copy of the document with all sections, see nature.com/documents/nr-reporting-summary-flat.pdf

Life sciences study design

All studies must disclose on these points even when the disclosure is negative.

Sample size	No sample size calculation was performed, sample sizes were chosen based on previous experience and on what is common practice in the field to study essential components of this signaling cascade.<br><br>Compare: Hrdinka et al. https://doi.org/10.1016/j.celrep.2016.02.062 or Hrdinka et al. https://doi.org/10.15252/embj.201899372 or Stafford et al. https://doi.org/10.1016/j.celrep.2018.01.024
Data exclusions	No data were excluded from the analysis.
Replication	All experiments were independently repeated as indicated in the respective figure legends. Furthermore, for most experiments, multiple cell lines were used to confirm reproducibility of the findings (KBM-7, THP-1, NOD2-HEK cells, HEK 293T and murine BMDMs).
Randomization	No randomization was necessary for this study, as there was no need to control for factors that would not be under direct experimental control.
Blinding	Blinding was not required for this study because no subjective analyses were performed that would have been biased by knowledge about the subjects studied.

Reporting for specific materials, systems and methods

We require information from authors about some types of materials, experimental systems and methods used in many studies. Here, indicate whether each material, system or method listed is relevant to your study. If you are not sure if a list item applies to your research, read the appropriate section before selecting a response.

Materials & experimental systems

n/a	Involved in the study	
☐	☒	Antibodies
☐	☒	Eukaryotic cell lines
☒	☐	Palaeontology and archaeology
☐	☒	Animals and other organisms
☒	☐	Human research participants
☒	☐	Clinical data
☒	☐	Dual use research of concern

Methods

n/a	Involved in the study	
☒	☐	ChIP-seq
☐	☒	Flow cytometry
☒	☐	MRI-based neuroimaging

Antibodies

Antibodies used

rabbit anti-NAGK (ab203900, Abcam)
rabbit anti-RIPK2 (#4142, Cell Signaling Technology)
mouse anti-β-actin HRP (sc-47778, Santa Cruz Biotechnology)
rabbit anti-phospho p65 (#3033, Cell Signaling Technology)
rabbit anti-phospho p38 (#9211, Cell Signaling Technology)
mouse anti-phospho IκBα (#9246, Cell Signaling Technology)
mouse anti-ubiquitin (#3936, Cell Signaling Technology)
anti-mouse IgG HRP linked (#7076, Cell Signaling Technology)
anti-rabbit IgG HRP (#7074, Cell Signaling Technology).

Validation

With the exception of the anti-NAGK antibody, we did not empirically validate the antibodies ourselves, but appropriate controls were performed to ensure that appropriate conclusions were drawn. All antibodies were purchased from commercial suppliers and the corresponding validation studies can be found on their websites:

rabbit anti-RIPK2 (#4142, Cell Signaling Technology)
https://www.cellsignal.com/products/primary-antibodies/rip2-d10b11-rabbit-mab/4142

mouse anti-β-actin HRP (sc-47778, Santa Cruz Biotechnology)
https://www.scbt.com/p/beta-actin-antibody-c4

rabbit anti-phospho p65 (#3033, Cell Signaling Technology)
https://www.cellsignal.com/products/primary-antibodies/phospho-nf-kb-p65-ser536-93h1-rabbit-mab/3033

rabbit anti-phospho p38 (#9211, Cell Signaling Technology)
https://www.cellsignal.com/products/primary-antibodies/phospho-p38-mapk-thr180-tyr182-antibody/9211

mouse anti-phospho IκBα (#9246, Cell Signaling Technology)
https://www.cellsignal.com/products/primary-antibodies/phospho-ikba-ser32-36-5a5-mouse-mab/9246

mouse anti-ubiquitin (#3936, Cell Signaling Technology)
https://www.cellsignal.com/products/primary-antibodies/ubiquitin-p4d1-mouse-mab/3936

anti-mouse IgG HRP linked (#7076, Cell Signaling Technology)
https://www.cellsignal.com/products/secondary-antibodies/anti-mouse-igg-hrp-linked-antibody/7076

anti-rabbit IgG HRP (#7074, Cell Signaling Technology)
https://www.cellsignal.com/products/secondary-antibodies/anti-rabbit-igg-hrp-linked-antibody/7074

Eukaryotic cell lines

Policy information about cell lines

Cell line source(s)

KBM-7 cells were from Thijn Brummelkamp.
THP-1 cells were from DSMZ (ACC 16).
HEK 293T cells were from DSMZ (ACC 635).
NOD2-HEK cells (HEK-Blue™-NOD2 cells) were from Invivogen.

Authentication

Cell lines were not additionally authenticated.

Mycoplasma contamination

All cell lines were tested negative for mycoplasma contamination at the beginning of the study, but were not tested routinely thereafter.

Commonly misidentified lines
(See ICLAC register)

No commonly misidentified cell lines were used in this study.

Animals and other organisms

Policy information about studies involving animals; ARRIVE guidelines recommended for reporting animal research

Laboratory animals	1 female and 2 male WT mice (C57BL/6J) and 2 female and 1 male Nagk-/- mice (C57BL/6J) (all littermates) aged 12 weeks were used for BMDM isolation.<br><br>All mice were housed in standard cages in a specific pathogen-free facility (21±1°C, on a 12-h light/dark cycle, with average humidity of around 55%) with ad libitum access to food and water in the animal facility at the Centre for Neuropathology.
Wild animals	No wild animals were used in this study.
Field-collected samples	No field-collected samples were used in this study.
Ethics oversight	All mice were handled according to institutional guidelines approved by the animal welfare and use committee of the government of Upper Bavaria.

Note that full information on the approval of the study protocol must also be provided in the manuscript.

Flow Cytometry

Plots

Confirm that:

☒ The axis labels state the marker and fluorochrome used (e.g. CD4-FITC).

☒ The axis scales are clearly visible. Include numbers along axes only for bottom left plot of group (a 'group' is an analysis of identical markers).

☒ All plots are contour plots with outliers or pseudocolor plots.

☒ A numerical value for number of cells or percentage (with statistics) is provided.

Methodology

Sample preparation	Cells were collected and washed with PBS. Cells were then passed through a 40-µm cell strainer and then vortexed before analysis.
Instrument	Cells were analysed on a BD LSR Fortessa and cells were sorted on a BD Fusion cell sorter (both BD Biosciences).
Software	Flow cytometry data were analysed using FlowJo 10.7.<br>For data collection the following software was used: BD FACSDiva 8.0.1 and 8.0.2.
Cell population abundance	Conclusions regarding cell population abundance were conducted when measuring the expression of the mScarlet reporter in KBM-7 cells. For these studies, a customised gate was drawn based on negative control samples (unstimulated controls) and then applied to all other samples of the respective data set.
Gating strategy	For analytical flow cytometry: Cells were first gated on FSC-A vs SSC-A to exclude debris. Subsequently, single cells were gated using the FSC-H vs FSC-W plot. Single cells were then analysed for fluorescence positivity by adjusting the threshold of the gate to non-fluorescent cells.<br>Cell sorting of fixed cells for mutation mapping: cells were identified using the FCS-A vs SSC-A blot to exclude debris. Subsequently, single cells were identified using DAPI-A vs DAPI-W blot. Single cells were displayed in a histogram, on which gating on 1n DNA content was performed to exclude diploid cells. Sorting was performed on the haploid cell population, the gates used for sorting were set to the bottom 4% IL1B-mScarlet-low and top 4% IL1B-mScarlet-high cells.

☒ Tick this box to confirm that a figure exemplifying the gating strategy is provided in the Supplementary Information.
